# Association between tooth loss and risk of oesophageal cancer: a dose–response meta-analysis

**DOI:** 10.1186/s40064-016-2711-6

**Published:** 2016-07-08

**Authors:** Yadong Wang, Juxiang Peng, Yan Li, Hong Luo, Guanglei Huang, Siyang Luo, Xinhai Yin, Jukun Song

**Affiliations:** Department of Oral and Maxillofacial Surgery, Guizhou Provincial People’s Hospital, Guizhou, 550002 China; Department of Orthodontics, Stomatology Hospital of Guiyang, Guizhou, 550002 China

## Abstract

Many epidemiological studies have found that tooth loss is associated with susceptibility to oesophageal cancer. However, a definitive answer is yet to be discovered, and the findings are inconclusive. We performed a meta-analysis to assess the relationship between tooth loss and oesophageal cancer risk. We searched PubMed and Embase databases to screen eligible studies up to June 2015. Nine observational studies (eight articles) involving 2604 patients and 113,995 participants were included in the meta-analysis. The combined odds ratio for tooth loss and oesophageal cancer was 1.53 (95 % CI 1.02–2.29) for the high versus lowest teeth loss categories. However, inconsistent results were detected in the stratified and sensitivity analysis. In dose–response analysis, the summary odds ratio for each one tooth loss increment was 1.01 (95 % CI 1.00–1.02). The current evidence, based solely on six case–control studies and three cohort studies, suggests that tooth loss is a potential marker of oesophageal cancer. However, no firm conclusion can be drawn at this time that tooth loss may play a causal role in development of oesophageal cancer. Additional large-scale and high-quality prospective studies are required to evaluate the association between tooth loss and risk of oesophageal cancer.

## Background

Oesophageal cancer, including squamous cell carcinoma and adenocarcinoma, is the seventh most common leading cause of cancer-related death in males in the United States, with an estimated 12,720 deaths in 2016, and one of the most common incident cancer, with an estimated 13,460 new cases (Siegel et al. 2016). Oesophageal cancer has been estimated to become a major concern with the rising trend of incidence in adult population. In each year, more than 450,000 people worldwide are diagnosed with the oesophageal cancer (Pennathur et al. 2013). The mortality from these cancers is high because most of the oesophageal cancer cases reported have been advanced at diagnosis (Napier et al. 2014). Therefore, finding and preventing the risk factors are important and significant in research. In the past decades, established risk factors for oesophageal cancer, including smoking tobacco, heavy alcohol drinking, poor diet (low fresh fruit and vegetable intake) and low socioeconomic status collectively account for less than half of all oesophageal cancer cases (Castellsague and Munoz 1997; Engel et al. 2003; Enzinger and Mayer 2003; Peng et al. 2016; Wang et al. 2012). The above mentioned data highlight the importance of screening patients who are at highest risk and identifying the potential risk factors for oesophageal cancer development.

Tooth loss significantly influences mastication, diets, nutrition intake, aesthetics, and food choice (Adegboye et al. 2012). Evidence from observational studies have suggested that tooth loss may be associated with oesophageal and gastric cancers (Abnet et al. 2008; Hiraki et al. 2008; Patel et al. 2013; Yin et al. 2016) and oral cancer (Wang et al. 2013; Zuo et al. 2015). Although tooth loss and oesophageal cancer share common risk factors, such as alcohol and tobacco use, it is unclear if tooth loss is a risk indicator for oesophageal cancer. Recently, a number of epidemiological studies have been conducted to examine the association between tooth loss and susceptibility to oesophageal cancer. However, the findings were mixed and inconsistent, with some of the studies reporting positive effects (Abnet et al. 2008; Hiraki et al. 2008; Patel et al. 2013) and others failing to demonstrate a significant association (Abnet et al. 2001, 2005a; Dar et al. 2013; Guha et al. 2007; Michaud et al. 2008). Although most of the studies included a very large number of potential subjects, the number of individual cases of oesophageal cancers was very small. Given the poor prognosis of oesophageal cancer and relatively small sample size of a single study, we aimed to summarise the association between tooth loss and risk of oesophageal cancer by conducting a meta-analysis. Clarifying this relationship may emphasise the importance of considering additional preventive methods for oesophageal cancer. The study was reported following the Preferred Reporting Items for Systematic Reviews and Meta-Analyses (PRISMA) Statement criteria (Moher et al. 2009).

## Methods

### Literature search

To identify all potentially eligible studies, a literature search was performed in PubMed and EMBASE databases for papers published from 1966 to June 2015 without restriction to regions, publication types, or languages. To identify eligible studies, the main search employed various combinations of Medical Subject Headings (MeSH) and non-MeSH terms: “esophageal cancer” OR “oesophageal cancer” OR “oesophageal neoplasms” OR “oesophageal squamous cell carcinoma” OR “oesophageal adenocarcinoma” and “tooth loss” OR “teeth loss”. References from eligible articles were also retrieved.

### Eligibility criteria

In the meta-analysis, the selected studies were considered eligible if they met the following inclusion criteria: (1) study design was either cohort, case control or cross-sectional studies; (2) the exposure was tooth loss; (3) the outcome was oesophageal cancer risk; (4) relative risk (RR) or odds ratio (OR) and hazard ratio (HR) with its 95 % confidence interval (CI) (or data to calculate these) were reported. Editorial letters, historical reviews and descriptive studies, such as case reports and case series, were excluded from the study. If the included population was duplicated in more than one study, only the most comprehensive study with the largest sample size was included. Two authors (SJK and YXH) independently assessed the inclusion of all retrieved studies and resolved any disagreements through discussion or after consultation with a third author (HGL).

### Data extraction

Two authors (SJK and YXH) independently extracted data from the selected studies using a standardised data extraction form. The following key points were collected: first author’s surname; year of publication; study design; country; duration of follow-up; sex; total number of cases and subjects; assessment methods for tooth loss; and multiple adjusted RR, OR and HR of tooth loss and corresponding 95 % CI for each category of exposure. The adjusted RR was extracted in preference to the non-adjusted RR; however, the unadjusted OR and CI were calculated when the OR was not provided. When more than one adjusted OR was reported, the ratio with the most number of adjusted variables was selected. Disagreements between reviewers regarding data extraction were resolved through discussion.

### Statistical analysis

The OR with 95 % CI was used as the common measure across all eligible studies. Because tooth loss caused oesophageal cancer was considered a rare event, the differences among estimates of relative risk were ignored and the HR and RR were directly converted to OR. A random-effects model of the DerSimonian and Laird method was used to calculate the summary risk estimates regardless of heterogeneity (DerSimonian and Laird 1986), which incorporates both within-study and between-study variabilities. Sensitivity analysis was performed to evaluate robustness and stability by sequentially omitting one study on each turn. Moreover, subgroup analysis was performed to explore the potential presence of heterogeneity and assess the influence of different inclusion criteria on the overall estimate.

The Newcastle-Ottawa Scale (NOS) was employed to evaluate the methodological quality of each study. Three major components were collected: selection of the study groups (0–4 points), ascertainment for the exposure of interest in the studies (0–3 points) and quality of the adjustment for confounding (0–2 points). The full score was nine stars, and the high-quality study was defined as a study with ≥5.

We also conducted a dose–response analysis using the method proposed by Greenland and Longnecker (1992). This method required that the distribution of cases and person-years or non-cases and risk estimates within the variance are known for at least three quantitative exposure categories. We explored a potential non-linear dose–response relationship between tooth loss and risk of oesophageal cancer using the generalised least squares for trend estimation and restricted cubic spline with four knots at 5, 35, 65 and 95 % of the distribution.

Publication bias was evaluated using Begg’s and Egger’s tests (rank correlation and linear regression methods, respectively) (Begg and Mazumdar 1994; Egger et al. 1997). All statistical analyses were carried out using Stata version 13.1 (StataCorp, College Station, TX, USA).

## Results

### Literature search and study characteristics

**Fig. 1:**
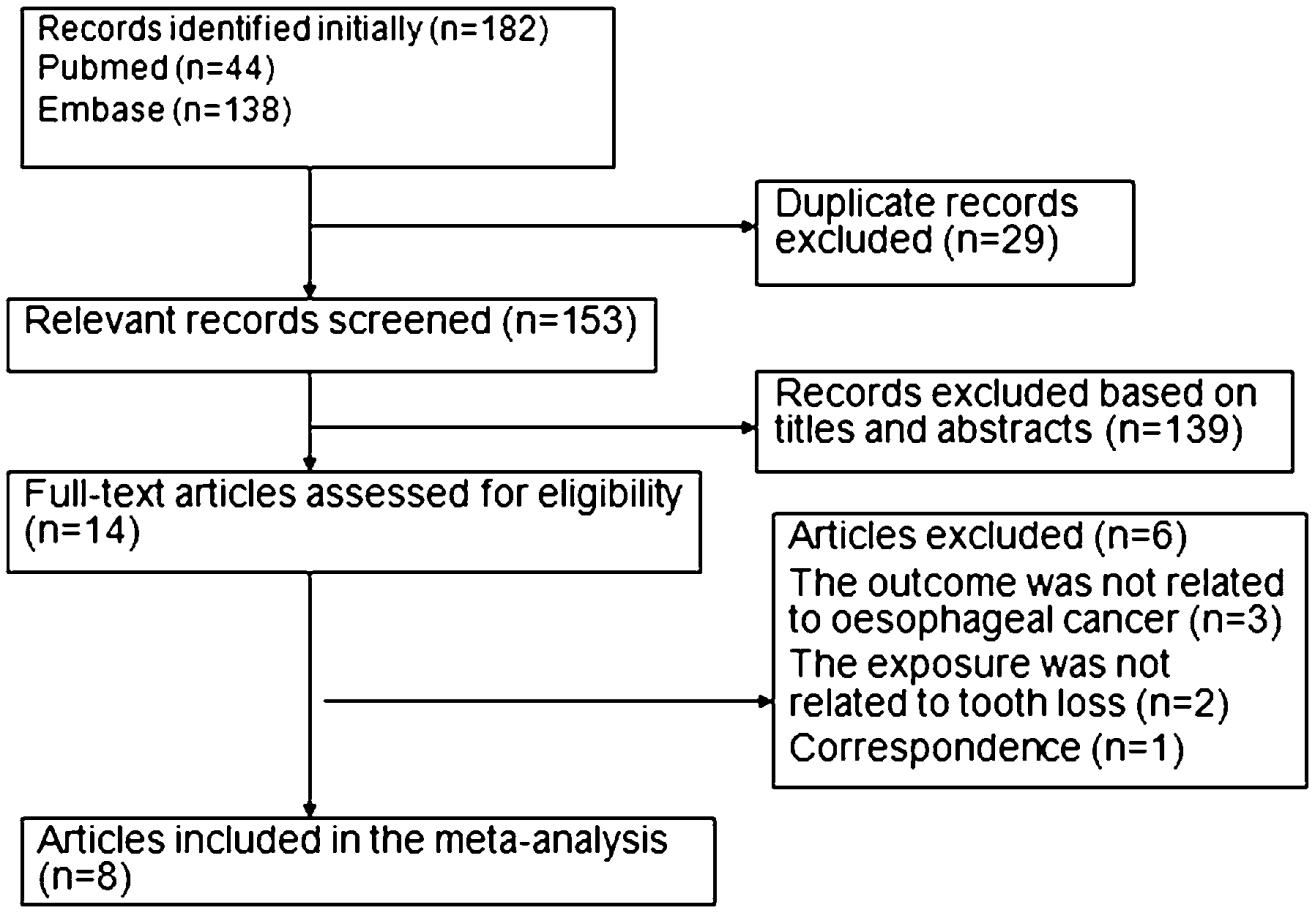
Flow chart from identification of eligible studies to final inclusion

A diagram showing the details of study inclusion is shown in Fig. 1. Using the outlined search strategy and selection based on the inclusion criteria, 182 studies were screened, 29 were excluded because they were duplicates and 139 were excluded based on their titles and abstracts. Fourteen full-text articles were reviewed for further assessment. One article was excluded because it was a correspondence (Conway 2009), and two articles were also excluded because the exposure was not related to tooth loss (Lee et al. 2014; Sepehr et al. 2005), three articles were subsequently excluded because the outcome was oesophageal squamous dysplasia (Dye et al. 2007; Wei et al. 2005) and upper gastrointestinal cancer (Abnet et al. 2005b). One article involved two case–control studies from central Europe and Latin America, so the article was regarded two studies (Guha et al. 2007). Finally, eight articles (nine studies) were considered eligible for inclusion in the meta-analysis (Fig. 1).

A total of eight articles (nine studies), six case–control studies (1804 cases and 5824 controls) and three cohort studies (800 oesophageal cancer cases and 106,367 participants) contributed to the analysis. The characteristics of the included case–control and cohort studies are listed in Table 1.

**Table 1:** Characteristic of studies included in the meta-analysis

Study	Year	Country	Study design	No. of subjects	No. of patients	Sex	Age, median (range), years	Assessment of tooth loss	Adjustment for covariates
Abnet	2001	China	A prospective cohort study	28,868	620	W and M	NA (40–69)	Questionnaire and clinical examination by interviewer	Adjusted for age sex, tobacco use, and alcohol use
Abnet	2005	Finland	A prospective cohort study	29,124	49	M	57.2 (50–69)	Questionnaire	Adjusted for age at randomization and education
Guha	2007	Central Europe	A hospital-based case–control study	1060	132	W and M	NA (NA)	Inspected by dentist or interviewer	Adjusted for age, sex, country/center, education, tobacco pack-years, cumulative alcohol consumption, and all other oral health variables
Guha	2007	Latin American	A hospital-based case–control study	1978	173	W and M	NA (NA)	Inspected by dentist or interviewer	Adjusted for age, sex, country/center, education, tobacco pack-years, cumulative alcohol consumption, and all other oral health variables
Abnet	2008	Iran	A population-based case–control study	843	283	W and M	65 (56–73)	Inspected by trained medical personnel	Adjusted for age, sex, place of residence, ethnicity, alcohol drinking, use of tobacco, opium, or both, education in three categories, number of appliances, and fruit and vegetable intake
Hiraki	2008	Japan	A hospital-based case–control study	1062	354	W and M	58.0 (20–79)	Self-administered questionnaire	Adjusted for age, sex, smoking and drinking status (never, former, current), vegetable and fruit intake, BMI, and regular exercise
Michaud	2008	United states	A prospective cohort study	48,375	131	M	NA (40–75)	Self-reported and clinical examination	Adjusted for age (continuous), race (White, Asian, Black), physical activity (quintiles), history of diabetes (yes/no), alcohol (quartiles), body mass index (<22, 22–24,9, 25–29.9, 30+), geographic location (South, West, Northeast, Midwest), height (quintiles), calcium intake (quintiles), total caloric intake (quintiles), red meat intake (quintiles),fruit and vegetable intake (quintiles), and vitamin D score (deciles) smoking history (never, past quit ≤10 years, past quit >10 years, current 1–14 cigarettes per day, 15–24 cigarettes per day, 25+ cigarettes per day), and pack-years (continuous)
Dar	2013	India	A case–control study	2367	703	W and M	Case: 61.6; Control: 59.8	Inspected by dentist	Adjusted for age, ethnicity, residence, education, wealth score, fruit and vegetable intake, bidi smoking, gutka chewing, alcohol consumption andcumulative use of hookah, cigarette, and nass
Patel	2013	Kenya	A hospital-based case–control study	318	159	W and M	56.1 (NA)	Questionnaire	Unadjust

*NA* not available, *M* male, *W* female

The eligible articles were published from 2001 to 2013. The number of oesophageal cancer patients ranged from 49 to 620 in the cohort studies and from 132 to 703 in the case–control studies. Four studies were conducted in Asia (Abnet et al. 2001, 2008; Dar et al. 2013; Hiraki et al. 2008), two in Europe (Abnet et al. 2005a; Guha et al. 2007), one in North America (Michaud et al. 2008), one in Africa (Patel et al. 2013) and one in Latin America (Guha et al. 2007). In all articles, cases were histologically, pathologically or clinically confirmed as oesophageal cancer and clearly showed the endpoint assessment of the diagnostic criteria. However, tooth loss was assessed using different strategies. Three articles used a questionnaire to classify tooth loss (Abnet et al. 2005a; Hiraki et al. 2008; Patel et al. 2013), whereas clinical examination was used as a diagnostic criteria in four articles (Abnet et al. 2001, 2008; Dar et al. 2013; Guha et al. 2007). The other one article was a self-report study (Michaud et al. 2008).

Five articles reported OR (Abnet et al. 2008; Dar et al. 2013; Guha et al. 2007; Hiraki et al. 2008; Patel et al. 2013), two reported HR (Abnet et al. 2005a; Michaud et al. 2008) and the other one reported RR (Abnet et al. 2001). One article was exclusive to men (Michaud et al. 2008), whereas the remaining studies included both men and women (Abnet et al. 2001, 2005a, 2008; Dar et al. 2013; Guha et al. 2007; Hiraki et al. 2008; Patel et al. 2013). One article did not adjust for confounding factors (Patel et al. 2013), but the other seven articles adjusted to various risk factors for oesophageal cancer, such as age, sex and education (Abnet et al. 2001, 2005a, 2008; Dar et al. 2013; Guha et al. 2007; Hiraki et al. 2008; Michaud et al. 2008). In addition, six articles controlled adjusted values, such as smoking and alcohol drinking (Abnet et al. 2001, 2008; Dar et al. 2013; Guha et al. 2007; Hiraki et al. 2008; Michaud et al. 2008).

We used NOS to evaluate the quality of the eligible studies (Table 2), in which the median NOS score was 6.5 (range of 4–8).

**Table 2:** Quality assessment of included studies based on Newcastle-Ottawa scale

Author	Year	Selection	Comparability	Exposure
Abnet	2001	3	1	2
Abnet	2005	3	2	2
Guha	2007	3	2	3
Abnet	2008	3	1	2
Hiraki	2008	3	2	2
Michaud	2008	3	1	3
Dar	2013	3	1	2
Patel	2013	2	0	2

### Risk of tooth loss on oesophageal cancer events

**Fig. 2:**
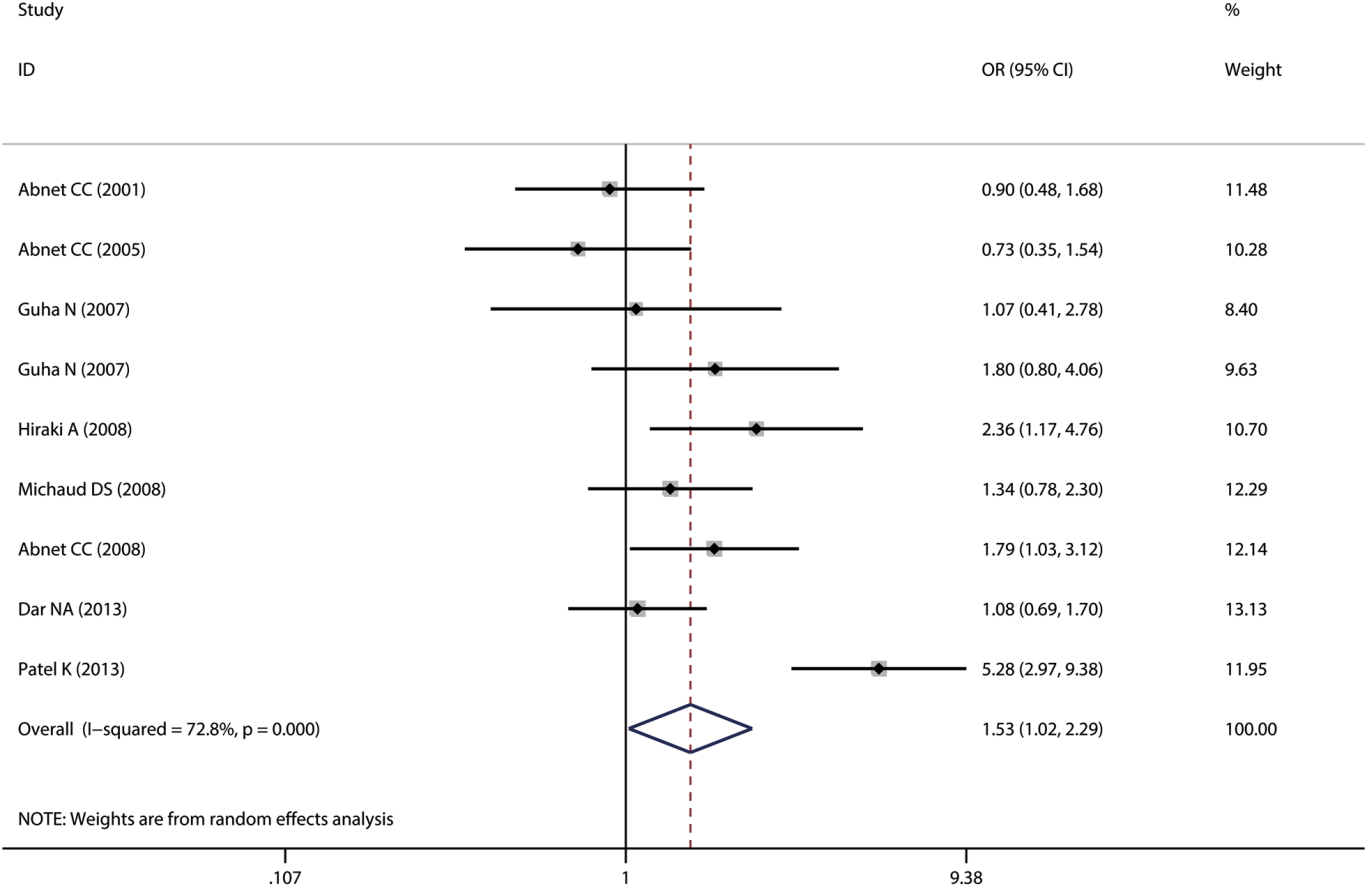
Forest plot of tooth loss and risk of oesophageal cancer. Studies are pooled with a random-effects model

The meta-analysis showed that compared with the lowest category, tooth loss was associated with 53 % higher rate in the highest group (OR 1.53, 95 % CI 1.02–2.29), and a significant heterogeneity was detected (*I*^2^ = 72.8 %, heterogeneity P = 0.000) (Fig. 2). In sensitivity analysis, the unstable results for oesophageal cancer risk was observed, which ranged from 1.29 (95 % CI 1.00–1.67) with low heterogeneity (*I*^2^ = 23.3 %, P_for heterogeneity_ = 0.244) [excluding the study by Patel et al. (2013)] to 1.67 (95 % CI 1.10–2.53) with significant heterogeneity (*I*^2^ = 72.2 %, P_for heterogeneity_ = 0.001) [excluding the study by Abnet et al. (2005a)]. When stratifying the data into subgroups based on different exclusion criteria, the results are significantly inconsistent (Table 3).

**Table 3:** Summary of results

	Studies, N	OR (95 % CI)	P value	P of heterogeneity	*I* ^2^ (%)
Total	9	1.53 (1.02–2.29)	0.040	0.000	72.8
Country					
Asia	4	1.38 (0.92–2.07)	0.118	0.112	49.9
Europe	2	0.84 (0.47–1.52)	0.570	0.536	0.0
America	1	1.34 (0.78-2.30)	0.289	NA	NA
Latin America	1	1.80 (0.80–4.06)	0.157	NA	NA
Africa	1	5.28 (2.97–9.38)	0.000	NA	NA
Effect size					
OR	6	1.93 (1.14–3.25)	0.014	0.001	74.8
HR	2	1.05 (0.58-1.88)	0.880	0.196	40.3
RR	1	0.90 (0.48–1.68)	0.740	NA	NA
Sample size					
Large	5	1.84 (0.99–3.42)	0.055	0.000	83.0
Small	4	1.19 (0.84–1.71)	0.329	0.410	0.0
Adjustment for smoking and alcohol drinking					
Yes	7	1.36 (1.06–1.74)	0.014	0.350	10.4
No	2	1.99 (0.29–13.85)	0.486	0.000	94.1
NOS score					
High	8	1.29 (1.00–1.67)	0.053	0.244	23.3
Low	1	5.28 (2.97–9.38)	0.000	NA	NA
Assessment of tooth loss					
Inspected by dentists or interviewers	5	1.25 (0.95–1.64)	0.113	0.413	0.0
Questionnaires	3	2.13 (0.69–6.58)	0.190	0.000	88.3
Self-reported	1	1.34 (0.78–2.30)	0.289	NA	NA
Study design					
Case control study	6	1.93 (1.14–3.25)	0.014	0.001	74.8
Cohort	3	1.02 (0.71–1.46)	0.910	0.385	0.0

*OR* odds ratio, *CI* confidence interval, *NA* not available, *Large* ≥100 cases, *Small* <100 cases, *High* NOS score of ≥5, *Low* NOS score of <5

### Dose–response meta-analysis

**Fig. 3:**
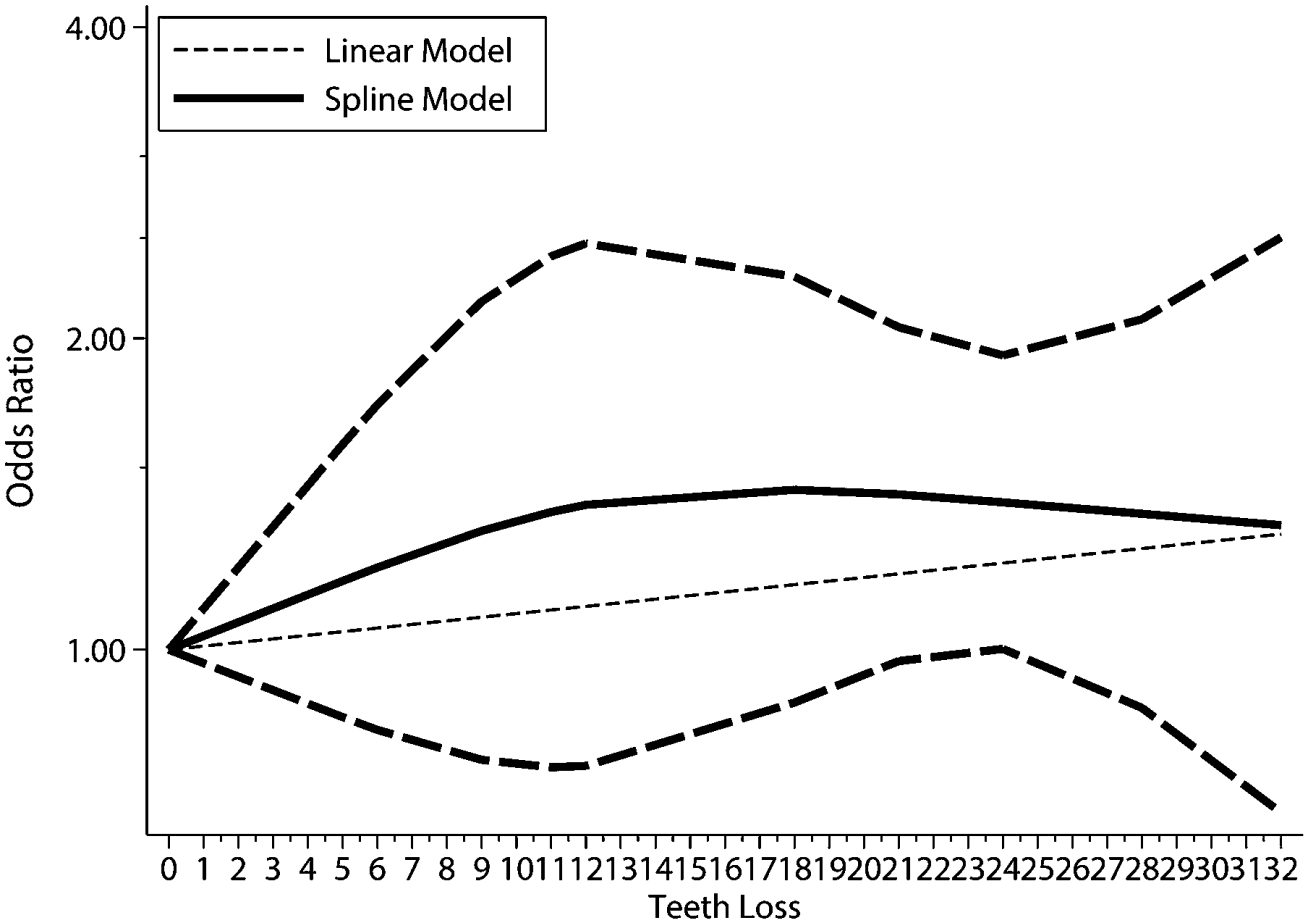
Dose–response analysis of each one tooth loss increment and risk of oesophageal cancer

Five studies (six articles) were included in the dose–response association between tooth loss and risk of oesophageal cancer, with a total of 1421 cases and 81,828 participants (Abnet et al. 2005a; Dar et al. 2013; Guha et al. 2007; Hiraki et al. 2008; Michaud et al. 2008). In dose–response analysis, the summary OR for loss of each one tooth loss increment was 1.01 (95 % CI 1.00–1.02), and no evidence of nonlinear relationship was observed (P for nonlinearity test = 0.08; Fig. 3).

### Publication bias

**Fig. 4:**
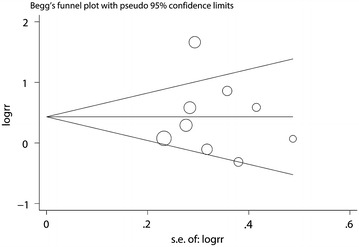
Begg’s funnel plot for publication bias analysis for tooth loss and risk of oesophageal cancer

Both Begg’s test and Egger’s funnel plot asymmetry test (rank correlation test and regression method, respectively) in the meta-analysis indicated no significant publication bias (Begg’s test, P = 0.917; Egger’s test, P = 0.920; Fig. 4).

## Discussion

To the best of our knowledge, this meta-analysis is the first to explore the association between tooth loss and risk of oesophageal cancer. The pooled results from the meta-analysis of nine observational studies (eight articles) using a random effects model revealed positive association between tooth loss and risk of oesophageal cancer.

Results from subgroup analyses indicated country, effect size, sample size, adjustment for smoking and alcohol drinking, quality of NOS scale, assessment of tooth loss and study design might be potential sources of heterogeneity. Despite of intrinsic limitations of observational study, some results from subgroup analyses remain notable. In subgroup analyses for study design, when we restricted the analysis to the six case–control studies and three cohort studies, the summary OR of any fracture for the highest category of tooth loss versus lowest category were 1.93 (95 % CI 1.14–3.25) and 1.02 (95 % CI 0.71–1.46). The combined OR for oesophageal cancer was 0.84 (95 % CI 0.47–1.52) for studies conducted in Europe, and 1.38 (95 % CI 0.92–2.07) in Asia. Furthermore, smoking and alcohol consumption is considered to be significant and dependent risk factor for oesophageal cancer risk. So, we also performed subgroup analyses among studies adjusted for smoking and alcohol consumption. Only seven studies adjusted for smoking status, and the results showed tooth loss was associated with increased risk of oesophageal cancer (OR 1.36, 95 % CI 1.06–1.74), with low heterogeneity (*I*^2^ = 10.4 %, P_for heterogeneity_: 0.350). However, when stratified by sample size and assessment of tooth loss, a nonsignificant association was detected.

Evidence from observational studies shows that tooth loss has been associated with multiple adverse health effects including epilepsy (Karolyhazy et al. 2005), cardiovascular disease (CVD) (Joshipura et al. 1998; Lowe et al. 2003; Watt et al. 2012), cognitive impairment (Luo et al. 2015; Peres et al. 2015; Zhu et al. 2015), and cancer (Idrissi Janati et al. 2016; Yin et al. 2016). However, no definitive mechanisms were established between tooth loss and cancer (Fitzpatrick and Katz 2010; Meyer et al. 2008). Tooth loss is a marker of systemic inflammation (Buchwald et al. 2013). The scientific rationale behind the potential association is that inflammation is a major factor in both tooth loss and cancer (Coussens and Werb 2002; Karin et al. 2006; van Kempen et al. 2006). Furthermore, the oral cavity, which provides a gateway between the external environment and the esophagus/gastrointestinal tract, functions in food ingestion and digestion. Tooth loss is also related to poorer oral hygiene (Adegboye et al. 2012; Marshall et al. 2002). Poorer oral hygiene potentially affects the gastrointestinal flora and nutritional status and may thus have implications for the development of cancer (Huang et al. 2016; Oji and Chukwuneke 2012).

This meta-analysis presents several limitations that must be considered in interpreting the results. Firstly, case–control studies have intrinsic limitations, such as selective bias and recall or memory bias. This limitation can partly explained the different results between case–control and cohort studies in the stratified analysis. Secondly, although the meta-analysis was based on a large number of participants with only nine studies included and was devoid of interventional studies, the combined estimates remained questionable. Thirdly, although we selected the highest multivariable-adjusted effect estimates in the meta-analysis, we cannot neglect the effect of residual confounding factors, such as diabetes, gastroesophageal reflux, and socioeconomic status. Fourthly, a significant heterogeneity was detected. Heterogeneity among studies should not be ignored even if it is highly common in a meta-analysis. Studies included in this meta-analysis are heterogeneous in terms of different populations investigated and diagnostic criteria for tooth loss, thereby contributing to the heterogeneity in the pooled analysis. Furthermore, unstable results were observed in subgroup and sensitivity analysis, which indicated that more relevant articles are needed to further explore this association. Fifthly, tooth loss assessment varied among studies. Clinical examination was used in four articles to classify tooth loss. A questionnaire was used as diagnostic criteria of tooth loss in three articles whereas one article was self-reported. The findings are likely to be influenced by misclassification of exposure because the majority of studies employed different methods to assess and categorize tooth loss. Therefore, the results should be considered with caution because of exposure misclassification. Overall, these limitations may affect our final conclusions.

In conclusion, our meta-analysis indicates that tooth loss is a potential marker of oesophageal cancer, suggesting that people who have lost teeth should pay attention to the symptoms for oesophageal cancer. However, we can not concluded at this time that tooth loss may be a casual factor for oesophageal cancer due to significant heterogeneity among studies and mixed results between case–control studies and cohort studies. Additional large-scale and high-quality prospective studies are required to evaluate the association between tooth loss and risk of oesophageal cancer.
